# KCN1, a Novel Synthetic Sulfonamide Anticancer Agent: *In Vitro* and *In Vivo* Anti-Pancreatic Cancer Activities and Preclinical Pharmacology

**DOI:** 10.1371/journal.pone.0044883

**Published:** 2012-09-13

**Authors:** Wei Wang, Lin Ao, Elizabeth R. Rayburn, Hongxia Xu, Xiangrong Zhang, Xu Zhang, Subhasree Ashok Nag, Xuming Wu, Ming-Hai Wang, Hui Wang, Erwin G. Van Meir, Ruiwen Zhang

**Affiliations:** 1 Department of Pharmaceutical Sciences, School of Pharmacy, Texas Tech University Health Sciences Center, Amarillo, Texas, United States of America; 2 Cancer Biology Center, School of Pharmacy, Texas Tech University Health Sciences Center, Amarillo, Texas, United States of America; 3 Division of Clinical Pharmacology, Department of Pharmacology and Toxicology, University of Alabama at Birmingham, Birmingham, Alabama, United States of America; 4 Nantong Cancer Hospital, Nantong University, Nantong, China; 5 Department of Biomedical Sciences, Texas Tech University Health Sciences Center, Amarillo, Texas, United States of America; 6 Institute for Nutritional Sciences, Shanghai Institutes for Biological Sciences, Chinese Academy of Sciences, Shanghai, China; 7 Department of Neurosurgery, Emory University School of Medicine, Atlanta, Georgia, United States of America; 8 Department of Hematology and Medical Oncology, Emory University School of Medicine, Atlanta, Georgia, United States of America; 9 Winship Cancer Institute, Emory University, Atlanta, Georgia, United States of America; Wayne State University School of Medicine, United States of America

## Abstract

The purpose of the present study was to determine the *in vitro* and *in vivo* anti-cancer activity and pharmacological properties of 3,4-dimethoxy-*N*-[(2,2-dimethyl-*2H*-chromen-6-yl)methyl]-*N*-phenylbenzenesulfonamide, KCN1. In the present study, we investigated the *in vitro* activity of KCN1 on cell proliferation and cell cycle distribution of pancreatic cancer cells, using the MTT and BrdUrd assays, and flow cytometry. The *in vivo* anti-cancer effects of KCN1 were evaluated in two distinct xenograft models of pancreatic cancer. We also developed an HPLC method for the quantitation of the compound, and examined its stability in mouse plasma, plasma protein binding, and degradation by mouse S9 microsomal enzymes. Furthermore, we examined the pharmacokinetics of KCN1 following intravenous or intraperitoneal injection in mice. Results showed that, in a dose-dependent manner, KCN1 inhibited cell growth and induced cell cycle arrest in human pancreatic cancer cells *in vitro*, and showed *in vivo* anticancer efficacy in mice bearing Panc-1 or Mia Paca-2 tumor xenografts. The HPLC method provided linear detection of KCN1 in all of the matrices in the range from 0.1 to 100 µM, and had a lower limit of detection of 0.085 µM in mouse plasma. KCN1 was very stable in mouse plasma, extensively plasma bound, and metabolized by S9 microsomal enzymes. The pharmacokinetic studies indicated that KCN1 could be detected in all of the tissues examined, most for at least 24 h. In conclusion, our preclinical data indicate that KCN1 is a potential therapeutic agent for pancreatic cancer, providing a basis for its future development.

## Introduction

Cancer remains a major public health problem worldwide. There are increasing pre-clinical and clinical discoveries that have bettered the prognosis for patients diagnosed with cancers, especially breast and prostate cancers. In contrast, there have been only minimal improvements in the outcome for patients with pancreatic cancer. Pancreatic cancer is characterized by its invasive nature, ability to evade aggressive therapy, and frequent late stage diagnosis [1]. The mortality rate for pancreatic cancer remains high, with an average survival of only <10 months after diagnosis [1]–[4]. There is an urgent need for the development of novel effective and safe agents for the treatment of pancreatic cancer.

**Figure 1:**
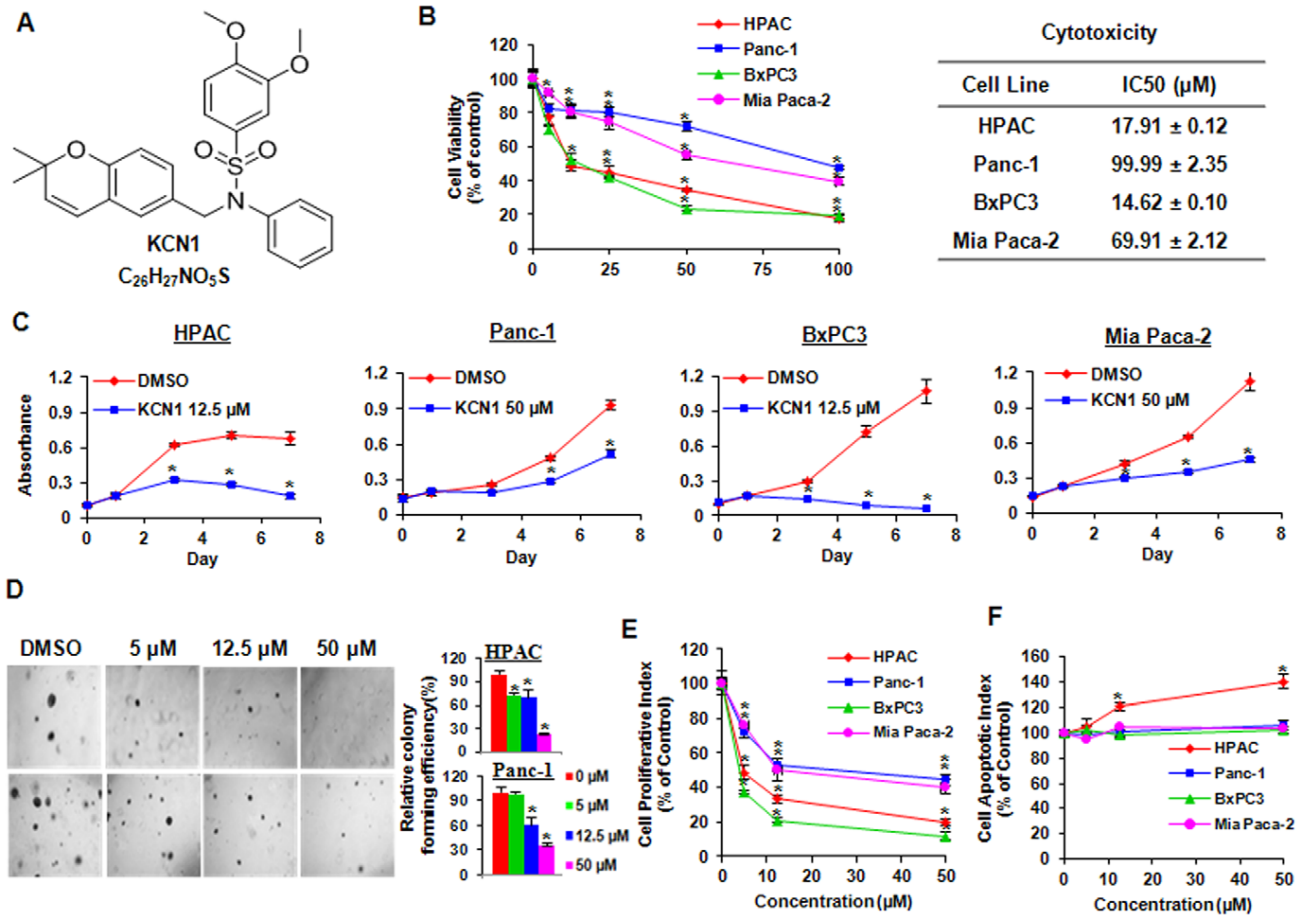
*In vitro* anticancer activities of KCN1 against pancreatic cancer cells. (A) Chemical structure of the KCN1. (B) Cell growth inhibitory activity of KCN1 in human pancreatic cancer cells. Cells were exposed to various concentrations of KCN1 for 72 h, followed by an MTT assay. (C) Cell growth inhibitory activity of KCN1 in a time-dependent manner. Cells were exposed to KCN1 for different time points, followed by an MTT assay. (D) Inhibition of anchorage-independent growth by KCN1 in pancreatic cancer cells. Cells were treated with various concentrations of KCN1 in soft agar. The cultures were maintained in the incubator for two weeks, then cell colonies were observed and scored under a microscope. (E) Anti-proliferative effect of KCN1 on human pancreatic cancer cells. Cells were exposed to various concentrations of KCN1 for 24 h, followed by measurement of cell proliferation using the BrdUrd assay. The proliferation index was calculated against untreated control cells. (F) Apoptotic effect of KCN1 on human pancreatic cancer cells. Cells were exposed to various concentrations of KCN1 for 48 h, followed by measurement of apoptosis by an Annexin V assay. The apoptotic index was calculated against untreated control cells. All assays were performed in triplicate. (^#^p<0.05, *p<0.01).

We have been interested in developing novel cancer therapeutic agents for human cancers with no current effective treatment, such as brain tumor and pancreatic cancer. A unique feature of solid cancers is that their rapid growth often results in reduced oxygen availability due to the formation of inadequate or aberrant vasculature [5]. The hypoxic fraction of solid tumors is resistant to radiotherapy [6] and conventional chemotherapy [7]–[10], and hypoxia correlates with poor therapeutic outcome [7], [8], [11], [12]. At the molecular level, the transcription factor Hypoxia Inducible Factor-1 (HIF-1) has been identified as the key orchestrator of the biological response to hypoxia due to its transactivation of genes that are involved in many aspects of malignant tumor growth from cell survival and metabolism to angiogenesis and invasion [13]–[16]. The overexpression of HIF-1 results in the constitutive activation of its target pathways [13]–[18]. HIF-1 is a heterodimeric transcription factor consisting of two subunits, HIF-1α, which is oxygen-regulated, and HIF-1β, which is constitutively expressed. Several inhibitors targeting HIF-1α expression or its activities have been designed for the treatment of cancers; however, none of these compounds has yet been successful due to compound toxicity, limited activity, or poor pharmacological properties [18]–[25]. We have recently developed a novel synthetic aryl sulfonamide, termed KCN1 (Fig. 1A) that was initially thought to target HIF-1α pathway. However, in recent studies, KCN1 has been shown to exert its anti-cancer activities under both normoxic and hypoxic conditions in human glioma cancer cell lines [26]–[31]. Our subsequent mechanistic studies have indicated that KCN1 has significant HIF-1α-independent cytostatic activities. The present study was designed to determine the *in vitro* and *in vivo* anti-cancer activity of KCN1 in pancreatic cancer and its pharmacological properties.

Because the distribution and disposition of an agent within the body is vital for determining its efficacy and toxicity, early pharmacokinetic studies are of great importance for drug development [32], [33]. Such studies can provide information about the most effective route and frequency of administration, as well as an indication of how effective the agent will be against tumors at different sites within the body. They may also indicate possible sites of drug accumulation and/or toxicity [32], [33]. In the present study, we attempted to characterize the pharmacological properties of KCN1 in preclinical setting, with regard to its plasma stability, plasma protein binding, bioavailability, and distribution following systemic administration to mice. These results demonstrate the anti-tumor efficacy and favorable pharmacological properties of this novel agent, supporting its further development towards clinic trials.

## Results

### KCN1 has *in vitro* Anti-cancer Activity Against Pancreatic Cancer cells

#### Inhibition of cancer cell growth

The *in vitro* anti-cancer activity of KCN1 was assessed using the MTT assay. Four human pancreatic cancer cell lines (HPAC, Panc-1, BxPC3, and Mia Paca-2) were cultured with the compound at various concentrations ranging from 0–100 µM for 72 h, and cell viability was determined. The inhibitory effects of the compound on cell growth were illustrated in Fig. 1B. KCN1 inhibited cancer cell growth in a dose-dependent manner, accounting for 83% (P<0.01), 53% (P<0.01), 81% (P<0.01) and 61% (P<0.01) inhibition at 100 µM in the HPAC, Panc-1, BxPC3, and Mia Paca-2 cells, respectively. The Panc-1 cell line is known to express the Multidrug Resistance-Associated Protein 1 (MRP1) and is known to show resistance to several cancer therapeutic drugs. This might be the reason they were more resistant to KCN1 treatment compared with other cells. Fig. 1C showed the time-course of growth inhibition with KCN1 treatment in all four cell lines, suggesting the respective sensitivity to the compound as BxPC3> HPAC > Mia Paca-2>Panc-1. As shown in Fig. 1D, HPAC and Panc-1cells were suspended in soft agar and colonies were enumerated after 14 days of incubation of KCN1. KCN1 decreased the number of colonies formed in HPAC and Panc-1 cells by 4- and 3-fold, respectively.

#### Inhibition of cancer cell proliferation

The dose-dependent effect of KCN1 on cell proliferation was examined with a BrdUrd incorporation assay (Fig. 1E). The anti-proliferative effects were seen in all the four cell lines. At a concentration of 50 µM, KCN1 inhibited the proliferation by about 80% (P<0.01), 56% (P<0.01), 88% (P<0.01) and 60% (P<0.01) in the HPAC, Panc-1, BxPC3, and Mia Paca-2 cells, respectively. BxPC3 cells were more sensitive to KCN1 treatment at the highest concentration than the other three cell lines.

#### Effects on apoptosis

We also examinedwhetherKCN1 had an effect on cell apoptosis in pancreatic cancer cells (Fig. 1F). After a 48 h treatment, KCN1 showed negligible or weak apoptotic effects following exposure to a 50 µM concentration of the compound. KCN1 was able to induce apoptosis only in the HPAC cells (apoptotic index: 1.4-fold). The drug showed no detectable apoptotic activity in the other three cell lines. These results indicated that induction of apoptosis may not be the major anti-cancer mechanisms for KCN1.

#### Cell cycle arrest in G1 phase

In the four different pancreatic cancer cell lines, after 24 h treatment, KCN1 significantly induced cell cycle arrest in the G1 phase in a dose-dependent manner, with initial effects beginning at 5 µM in HPAC (P<0.01), BxPC3 (P<0.01), and Mia Paca-2 (P<0.01) cells, and 12.5 µM in Panc-1 (P<0.01) cells (Table 1). These results indicated that induction of cell cycle arrest may be a major anti-cancer mechanism for KCN1.

**Table 1:** Effects of KCN1 on cell cycle progression of pancreatic cancer cells.

KCN1	HPAC			BXPC3		
Conc.(µM)	G1	S	G2/M	G1	S	G2/M
**0**	39.4±3.6	35.2±1.8	25.7±3.1	45.1±3.2	25.6±2.2	29.3±2.9
**5**	62.1±4.9*	26.1±3.8	11.8±1.2	52.9±4.1*	20.8±3.8	26.4±1.3
**12.5**	66.8±1.6*	22.4±1.6	10.9±1.3	65.1±3.5*	17.5±2.1	17.4±1.9
**50**	68.8±2.7*	19.7±1.4	11.5±1.4	70.8±2.0*	13.7±0.9	15.5±1.7
**KCN1**	**Panc-1**			**Mia Paca-2**		
**Conc.(µM)**	**G1**	**S**	**G2/M**	**G1**	**S**	**G2/M**
**0**	39.5±1.6	29.6±0.1	30.9±1.4	46.7±2.3	19.9±2.1	33.4±2.5
**5**	40.3±2.2	28.8±3.2	30.9±0.8	50.4±3.6*	18.0±0.9	31.6±1.9
**12.5**	49.8±0.8*	21.4±0.9	28.8±1.6	55.2±1.2*	20.2±1.7	24.6±0.9
**50**	53.3±2.3*	17.6±0.9	29.1±1.4	58.9±3.9*	20.8±1.5	20.3±1.1

# P<0.05; *P<0.01 compared with the controls.

### KCN1 Modulates the Expression of Cell Cycle-related Proteins

**Figure 2:**
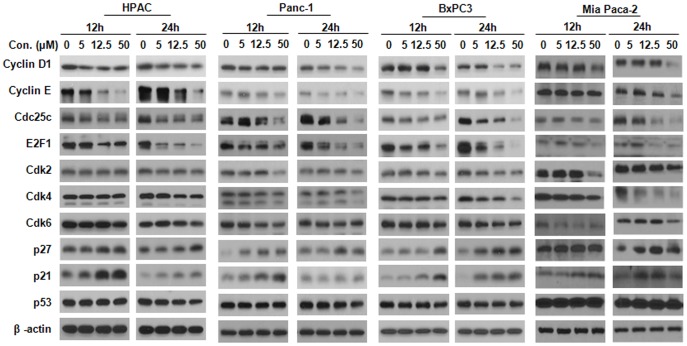
Effect of KCN1 on the expression of proteins related to cell cycle progression in human pancreatic cancer cells. HPAC, Panc-1, Mia Paca-2 and BxPC3 pancreatic cancer cells were exposed to several concentrations of the compound for 12 or 24 h, and then target proteins related to cell cycle progression were examined by Western blotting.

We investigated the possible mechanisms responsible for the anti-proliferative and cell cycle regulatory effects of KCN1 by evaluating its effects on the expression level of various proteins involved in regulating cell proliferation and cell cycle progression (Fig. 2). In all four cell lines, the treatment with KCN1 (12 h and 24 h) led to various levels of decreased expression of cell cycle regulators E2F1, Cdk2, Cdk4, Cdk6, Cdc25c, Cyclin D1, and Cyclin E. In contrast, exposure to KCN1 increased the expression of p21 and p27 in all four cell lines. These proteins are mainly involved in cell cycle progression and check-point control. These results further indicate that KCN1 exerts its anti-cancer activity through cell cycle arrest.

### KCN1 Inhibits the Growth of Xenograft Tumors

**Figure 3:**
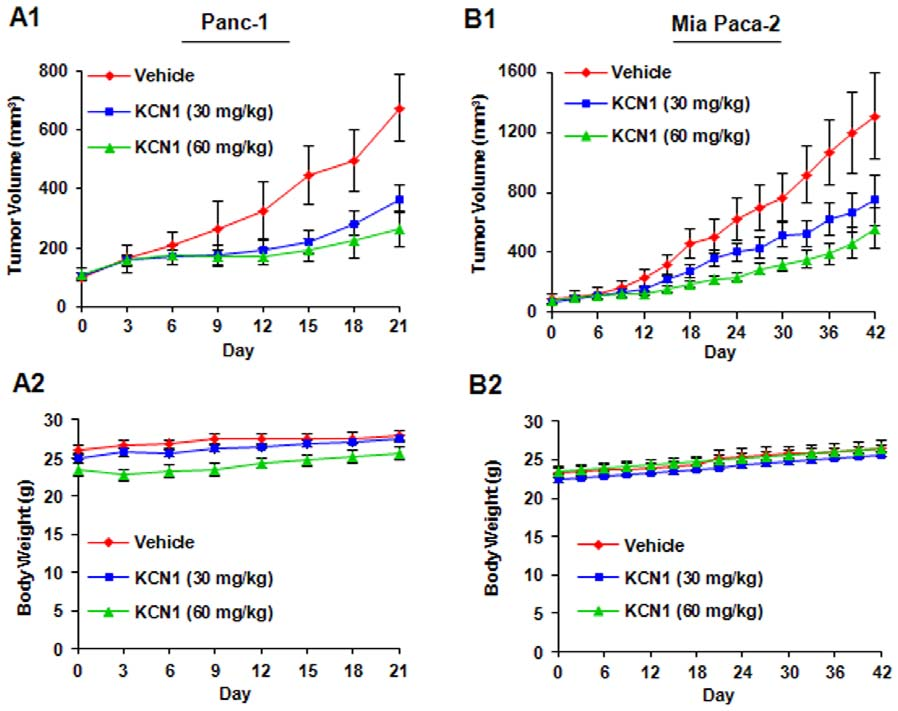
*In vivo* anticancer activity of KCN1 against pancreatic cancer cells. KCN1 was administered by i.p. injection to nude mice bearing Panc-1 (A1) or Mia Paca-2 (B1) xenograft tumors. KCN1 was administered by i.p. injection at doses of 30 and 60 mg/kg/d, 5 days/wk for 3 weeks for Panc-1 xenograft model and 6 weeks for Mia Paca-2 xenograft model, respectively. Control groups received vehicle only. Tumors volumes were measured every three days. Animals were also monitored for changes in body weight as a surrogate marker for toxicity when it was administered to nude mice bearing (A2) Panc-1 or (B2) Mia Paca-2 xenograft tumors.

To determine whether the compound was effective against *in vivo* tumors, we evaluated the anti-tumor effect of systemic delivery of KCN1 against the Panc-1 and Mia Paca-2 subcutaneous xenograft models in *nu/nu* mice. Systemic intraperitoneal (i.p.) treatment with KCN1 (30 or 60 mg/kg in a 1∶1 cremophor:ethanol formulation; 5 days/week) was initiated once the tumor volume reached ∼100 mm^3^_._ The compound significantly decreased the growth of the pancreatic xenograft tumors in a dose-dependent manner. In the Panc-1 xenograft model, tumor growth inhibition of approximately 46% (P<0.01) was observed at the 30 mg/kg dose and of 61% (P<0.01) at the 60 mg/kg dose on Day 21 (Fig. 3A1). Similar results were observed in the Mia Paca-2 xenograft model. This model appeared to be almost similarly sensitive to the drug, with the low dose (30 mg/kg) and high dose (60 mg/kg) decreasing tumor growth by about 43% (P<0.01) and 57% (P<0.01), respectively (Fig. 3B1). Additionally, there were no significant differences in body weights between controls and animals treated with KCN1 in both xenograft models (Figs. 3A2 and B2), indicating no obvious host toxicity at the therapeutic doses of KCN1.

### An HPLC Method for KCN-1 Analysis is Developed and Validated

**Figure 4:**
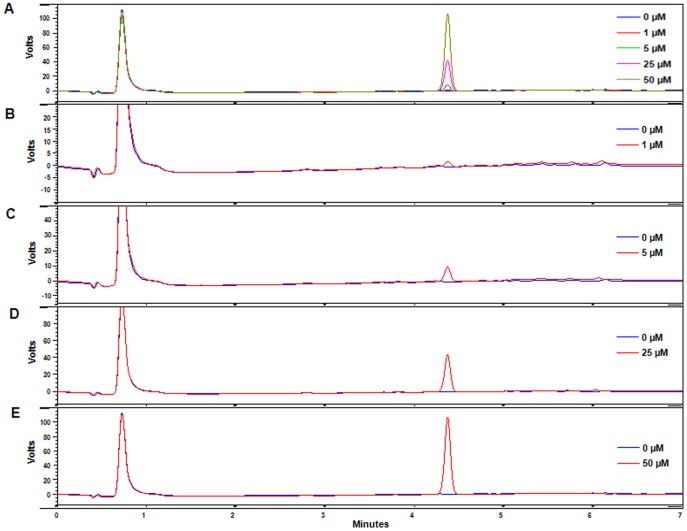
Representative chromatograms of KCN1. (A) KCN1 in control (drug-free) mouse plasma and mouse plasma spiked to contain 1, 5, 25, and 50 µM KCN1; A blank mouse plasma sample and mouse plasma sample spiked with 1 µM (B), 5 µM (C), 25 µM (D), and 50 µM (E) KCN1.

The HPLC method gave a linear calibration curve in mouse plasma for the investigated concentration range of 0.1–100 µM. The mean correlation coefficient (*r* ^2^) for daily calibration curves was  = 1.000. Calibration curves were also produced in homogenates of various mouse tissues, including the brain, skeletal muscle, kidneys, lungs, spleen, and heart, which had correlation coefficients of at least 0.992 for the same concentrations. The accuracy, precision, intra-day and inter-day variations were acceptable, with coefficients of variation (CV) between 4.25% and 12.62%, and the lower limit of detection (LOD) in plasma was 0.085 µM. The recovery of the compound in the various matrices ranged from ∼96% to ∼107%. Representative chromatograms of blank mouse plasma, control mouse plasma spiked with 1, 5, 25, and 50 µM KCN1 are shown in Figs. 4A–E. Similar chromatograms were obtained for the other tissues examined (data not shown). The specificity was demonstrated by the absence of any endogenous interference in the biological samples at the retention time of the peaks of KCN1.

### KCN1 is Stable in Mouse Plasma at Various Temperatures for Extended Durations

**Figure 5:**
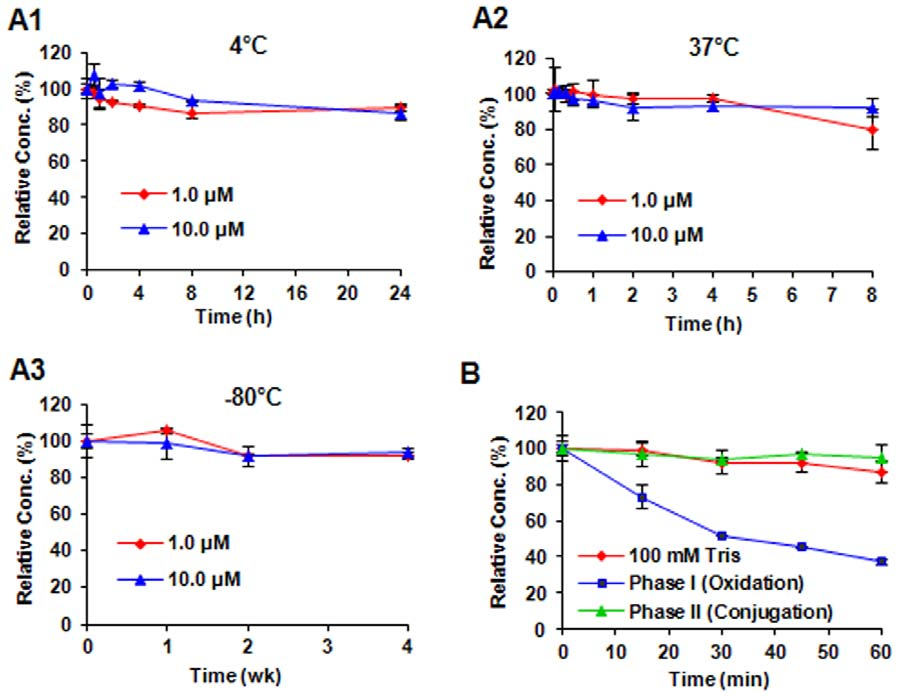
Stability and metabolism of KCN1. Stability of KCN1 in mouse plasma at 37°C (A1), 4°C (A2), and −80°C (A3). (B) Degradation of KCN1 by isolated mouse S9 liver fractions.

KCN1 was stable in mouse plasma at 37°C, with more than 80% of the compound remaining after an 8 hr incubation for both the low (1 µM) and high (10 µM) concentrations. We also found that KCN1 can be stored at 4°C for at least 24 hr with more than 90% of the compound remaining (Fig. 5A1), or at 37°C for at least 8 hr with more than 85% of the compound remaining (Fig. 5A2), or at −80°C for up to 4 wks with more than 92% of the compound remaining (Fig. 5A3).

### KCN1 Binds to Plasma Protein Extensively

KCN1 bound extensively to proteins in mouse plasma, with the low (1.0 µM) and high (10.0 µM) concentrations demonstrating 90.96% and 99.32% of the compound binding to plasma proteins, respectively.

### KCN-1 is Metabolized by S9 Enzymes (Phase I)

Because KCN1 appeared to be extensively metabolized or degraded in mouse plasma, we performed a preliminary study of the potential mechanism(s) by which KCN1 is metabolized using the S9 assay. This assay can be used to determine whether the compound is metabolized *via* phase I (NADPH-regenerating systems) or phase II (UDPGA and PAPS) enzymes extracted from mouse microsomes. The S9 studies indicated that KCN1 has a relatively long half-life in the presence of the phase II (conjugation) microsomal enzymes. However, there was a more than 60% decrease in the amount of KCN1 observed when it was incubated with mouse phase I (oxidation) enzymes (Fig. 5B), suggesting that it is likely metabolized by phase I enzyme(s).

### KCN1 has a Long Half-life and Wide Tissue Distribution in Mice after Intravenous and Intraperitoneal Administrations

**Figure 6:**
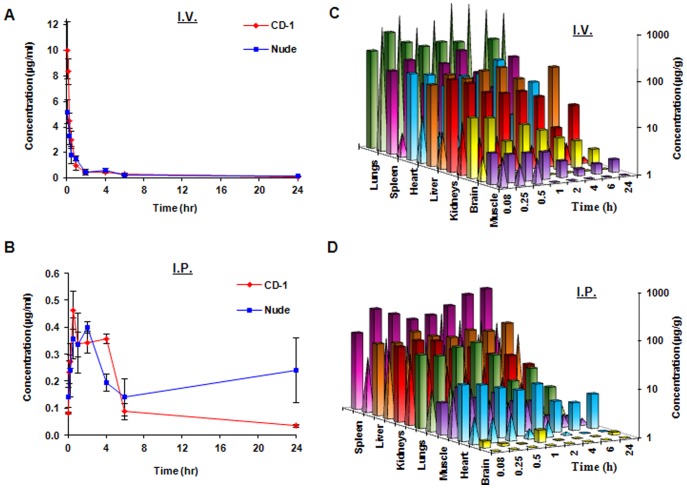
Distribution of KCN1 in mouse plasma and various tissues. Plasma concentration-time curves for KCN1 following (A) IV and (B) IP injection of 35 mg/kg in CD-1 and nude (nu/nu) mice. Time-dependent distribution of KCN1 in various tissues following (C) IV administration or (D) IP administration of 35 mg/kg of the compound. The columns represent nude mice, while the pyramids represent CD-1 mice.

Analysis of plasma samples collected from mice dosed with KCN1 (35 mg/kg) showed that plasma concentrations of the compound were still detectable for both strains at least 6 h after intravenous (I.V.) injections, and were detectable for even 24 h following intraperitoneal (I.P.) administration (Fig. 6A and 6B). Following bolus I.V. administration, the plasma concentrations were greater than 5 µg/mL at 5 minutes for both CD-1 (9.98 µg/mL) and nude mice (5.19 µg/mL). These levels declined to <0.3 µg/mL at 6 h after dosing in both strains. After I.P. dosing, the plasma levels reached their maximum at 30 min in CD-1 mice and at 2 h in nude mice. Overall, I.V. administration gave higher plasma AUC and C_max_ values, but lower T_1/2_ values compared to the I.P. route of administration (Table 2).

**Table 2:** Pharmacokinetic parameters of KCN1 following intravenous and intraperitoneal injection in CD-1 and nude mice.

Tissue	Strain	Route	C_max_	T_max_	AUC	T_1/2_	Cl
			(ng/ml)	(hr)	(hr·ng/ml)	(hr)	(ml/hr/kg)
Plasma	CD-1	IV	13.90	-	7.19	0.16	4.86
		IP	0.46	0.50	2.99	7.35	10.25
	Nu/nu	IV	6.76	-	5.88	0.60	5.95
		IP	0.40	2.00	5.00	2.65	5.91
Liver	CD-1	IV	6.14	0.08	58.44	1.15	563.33
		IP	65.56	4.00	748.11	3.50	39.12
	Nu/nu	IV	89.74	2.00	1480.49	3.39	19.15
		IP	44.75	0.50	881.33	2.52	33.73
Lungs	CD-1	IV	980.72	2.00	14177.52	23.28	1.18
		IP	10.01	0.25	54.26	2.27	571.31
	Nu/nu	IV	264.35	0.25	1858.44	2.00	15.99
		IP	44.97	1.00	242.18	1.04	141.14
Kidneys	CD-1	IV	13.49	0.08	18.78	18.78	368.38
		IP	28.85	0.08	113.97	2.50	288.24
	Nu/nu	IV	93.67	0.08	371.94	1.80	85.25
		IP	43.38	0.25	328.14	22.42	58.59
Spleen	CD-1	IV	3.17	0.08	25.33	0.46	1339.61
		IP	75.21	2.00	805.59	2.35	37.56
	Nu/nu	IV	113.17	2.00	1067.41	1.40	29.21
		IP	163.00	24.00	3180.37	0.81	10.39
Heart	CD-1	IV	10.29	0.08	24.36	22.08	771.20
		IP	4.48	0.17	18.95	2.19	1709.94
	Nu/nu	IV	88.39	4.00	603.96	0.36	56.71
		IP	14.65	0.08	122.72	1.22	265.80
Muscle	CD-1	IV	4.41	0.08	8.68	1.52	3954.09
		IP	9.41	0.17	49.09	1.86	663.45
	Nu/nu	IV	4.65	0.08	45.71	2.28	674.38
		IP	8.57	0.25	46.95	2.97	664.67
Brain	CD-1	IV	3.33	0.08	5.67	0.53	6085.91
		IP	0.34	1.00	3.03	3.13	10464.00
	Nu/nu	IV	19.49	0.08	97.24	3.38	322.49
		IP	1.80	1.00	24.14	0.95	1361.63

Cmax: maximum concentration of the compound observed; Tmax: time when the maximum concentration was observed; AUC: area under the concentration-time curve; T1/2: half-life of the compound; Cl: clearance.

Of interest, KCN1 could be detected in most of the sampled tissues (heart, lungs, liver, spleen, kidneys, and skeletal muscle; Fig. 6C and 6D) of both strains for at least 24 h after administration by either route. However, the compound could not be detected after 6 h in the brain of either strain of mice following either route of administration. Pharmacokinetic parameters were calculated for plasma and the various tissues (Table 2). The highest AUC values in both nude and CD-1 mice following I.V. injection were in the lungs, liver and spleen, and the tissues with the lowest AUC values following I.V. injection were the plasma, brain and skeletal muscle. The high concentration observed in the lungs after I.V. injection was likely due to accumulation of precipitated drug, although this was not verified experimentally. In both strains of mice, the spleen had the highest AUC following I.P. injection, followed by the liver. The plasma and brain had the lowest AUC values for both strains following I.P. injection.

Of the 35 mg/kg of KCN1 administered intravenously, less than 1% of the total KCN1 dose was recovered as parental KCN1 from the urine of both the CD-1 and nude mice during the first 24 hr, with less than 0.5% being recovered from both strains following I.P. injection. Approximately 1% of the initial dose of the parental compound was recovered in the feces of mice following I.P. injection, and less than 1% was recovered following I.V. injection (data not shown).

## Discussion

HIF pathway inhibitors represent a novel targeted therapeutic agent. Although success has been seen using agents targeting individual molecules (e.g. Herceptin, Gleevec), these agents are typically limited with regard to the types of cancer, and to sub-classes within the types of cancer, that can be treated. Because KCN1 inhibits a physiological process (hypoxia) that is inherent in the formation of tumors, rather than a cancer type-specific target, the agent can potentially be used to treat a variety of different cancers, including those for which there are currently no effective treatments, especially pancreatic cancer.

The test compound KCN1 was initially designed as an HIF-1α-inhibitor, but its inhibitory effects on cell proliferation and growth led us to speculate whether it might have an effect under normoxic condition. HIF-1α also plays an important role in the regulation of cell cycle. Under hypoxic conditions, it becomes stabilized leading to tumor survival *via* increased angiogenesis and anaerobic glycolysis. Surprisingly, HIF-1α also up-regulates genes that promote cell cycle arrest and apoptosis, such as p21, p27, p53 [34], [35]. In view of this, the benefits of HIF-1α inhibition in cancer therapy seem questionable as it can undo cell cycle arrest under hypoxic conditions and prevent cell death. These contradictory effects may limit the anti-cancer effect of HIF-1α inhibitors [34], [35]. However, if HIF-1α inhibitors have the additional effect of inducing cell cycle arrest or cell death, they might be better propositions as anti-cancer agents. Thus, it is imperative while developing HIF-1α inhibitors as anti-cancer agents to evaluate their effects on cell cycle and cell death.

The current study is the first to systematically investigate the *in vitro* and *in vivo* anti-tumor effects of KCN1 in pancreatic cancer cells in a HIF-1α-independent manner. We observed that KCN1 significantly decreased pancreatic cancer cell growth and proliferation, and led to cell cycle arrest under normoxic (20% O_2_) culture conditions. After evaluating the effects of the compound on *in vitro* proliferation, cell cycle progression and apoptosis, we concluded that cell cycle arrest was the main mechanism by which the compound exerts its cytostatic effects in cell culture. Cell cycle arrest is one of the most effective strategies for inhibiting tumor growth [36]. In the present study, we found that KCN1-mediated cell cycle arrest was achieved *via* a modulation of cyclin kinase inhibitor (CKI)- cyclin- cyclin-dependent kinase (CDK) machinery operating in the G1 phase of the cell cycle. A family of protein kinase complexes orchestrates cell cycle progression in eukaryotes [37]. Each complex is composed minimally of a catalytic subunit, the cdk, and its essential activating partner, the cyclin. Various combinations of cyclins and cdks control the cell cycle at different points [37]. For example, Cyclin E is expressed transiently during the G1/S transition and is rapidly degraded once the cell enters S phase [38]. Cyclin E regulates Cdk2 while Cyclin D1 regulates Cdk4 and Cdk6 [38]. These complexes are activated at various checkpoints after specific intervals during the cell cycle, but can also be induced and regulated by exogenous factors [37]–[39]. The cdks are subjected to inhibition by the binding of CKI such as the CIP/KIP (p21, p27) and INK4 families of proteins [37]–[39]. The transcription of genes required for the G1/S transition such as Cyclin E and Cyclin D1 is initiated by E2F1, which is under the control of the Rb tumor suppressor [37]–[39]. In this study, we examined the influence of KCN1 on the expression of several proteins known to be involved in these processes. In all four pancreatic cancer cell lines tested, we found decreased expression of cell cycleproteins, including E2F1, Cyclin D1, Cyclin E, Cdk2, Cdk4 and Cdk6, and increased expression of p21 and p27. While KCN1 induced cell cycle arrest in pancreatic cancer cell lines, it had little cytostatic effect on glioma cells and immortalized fibroblasts, suggesting that its effects might be cell type-dependent [31]. To confirm the therapeutic efficacy of the compound, it was also investigated for *in vivo* effects. KCN1 decreased the growth of both Panc-1 and Mia Paca-2 xenograft tumors.

Since there are no published reports about the pharmacokinetics, toxicity, or bioavailability of KCN1, we performed an evaluation of these pharmacological properties. We developed a suitable HPLC method for the detection of KCN1 in various biological matrices, and demonstrated that KCN1 is stable in mouse plasma (Fig. 5A1–A3), that it is extensively bound to plasma proteins, that it is likely metabolized by phase I enzyme(s) (Fig. 5B), and that it is distributed to various mouse tissues after both I.V. and I.P. administration (Fig. 6 and Table 2). Our initial pharmacology studies brought to light several interesting characteristics about KCN1. First, there was extensive accumulation of the compound in the lungs of mice following I.V. injection, and in the spleen and liver of mice following I.P. injection. Future studies are needed to determine the mechanisms for the unique distribution pattern of this drug. It was administered intraperitoneally at a dose of up to 60 mg/kg daily in a cremophor:ethanol formulation for up to 12 weeks, and this regimen was well tolerated by the animals. There were no apparent signs of toxicity in these animals, and their behavior and appearance were indistinguishable from control animals. Our preliminary analyses indicated that the values of blood cell populations and blood chemistry were within normal limits (data not shown). In addition, pathological examination of the major organs at necropsy demonstrated no ultra-structural changes in the brain, kidneys, GI tract or lungs (data not shown). Liver is the only organ where a treatment-related change was observed. Swelling was observed in the livers of animals at autopsy, tissue edema with bile duct stasis was indicated with pathological examination, but without any evidence of hepatocyte death. The observed swelling of the liver was reversed in the mice within 2–3 weeks after discontinuation of treatment, and may has been caused by the cremophor:ethanol formulation, which can interfere with hepatic blood flow [40]–[42].

The second point of interest is that there were differences in the pharmacokinetics of KCN1 between CD-1 and nude mice. While some of these differences may have been due to the normal variations between mice, differences in the time of year (season) when the studies were done, and differences due to the source of the mice (Harlan vs. Frederick), some of the differences between the strains of mice were still worth noting. For example, the CD-1 mice showed a much higher concentration in the lungs than the nude mice, despite receiving the same dose. However, in most cases, the nude mice showed a higher uptake of the agent to the various tissues examined (liver, kidneys, spleen, heart, skeletal muscle, and brain). There were also differences in the intraperitoneal bioavailability of KCN1, with the compound being absorbed much better following intraperitoneal injection in nude mice compared to CD-1 mice. This increased bioavailability likely was responsible for the higher tissue uptake. If similar results are found in repeated studies, it will be necessary to elucidate the mechanism(s) underlying these differences in uptake in order to determine whether such factors are likely to impact the pharmacokinetics of KCN1 in other species, including humans. Moreover, while CD-1 mice are frequently used to evaluate the pharmacokinetics of novel compounds, nude mice are the most commonly used mouse strain for anti-cancer efficacy studies. Differences in pharmacokinetics between the strains might lead to over- or under-estimation of the efficacy or toxicity of a compound.

We also noted that KCN1 apparently undergoes enterohepatic recirculation. Not only was the compound still present in many tissues, but in some cases it was higher at 24 h than at 4 or 6 h (e.g. liver and spleen). Additionally, KCN1 was still detectable at a low level for at least five days after intraperitoneal dosing (data not shown). These findings suggest that it may be possible to give KCN1 relatively infrequently, since the compound may remain in the target tissue for several days. However, it will be necessary to ensure that the compound does not exert toxic effects on the liver, gallbladder or other tissues exposed to high concentration of the compound for extended periods of time.

In addition to changes in the frequency of dosing for efficacy studies, changing the formulation may also improve the activity of the compound. Although the compound was effective when administered I.P. in cremophor:ethanol, the formulation is not ideal. More importantly, while KCN1 itself did not appear to exert any major toxicity, the vehicle led to liver swelling after several weeks of dosing in the control animals. Thus, changing the formulation might decrease the toxicity of the compound, and it is possible that optimizing the formulation and administration of the compound would also enhance its anti-cancer effects.

In summary, we have demonstrated that KCN1 can exert potent cytotoxicity and cell proliferation inhibition effects towards pancreatic cancer cells, and led to down-regulation of important oncogenic and pro-growth/pro-proliferation proteins. We have presented a valid method for detecting and quantifying KCN1 in various matrices. We also have presented initial pharmacokinetic data about the compound, indicating that it is well distributed, stable, and detectable in various tissues for a relatively long period of time. The information generated in this study will be useful for the further development of the compound.

## Materials and Methods

### Chemicals and Reagents

KCN1 was synthesized and purified as previously reported [29], [43]–[44], and the structure was confirmed by UV, IR, MS, and NMR spectroscopy. The purity of the compound was determined to be greater than 99%.

All chemicals and solvents used for sample preparation and high-performance liquid chromatography (HPLC) analysis were of analytical grade. Methanol (HPLC grade) was purchased from Fisher Chemicals (Fairlawn, NJ) and triethylamine was purchased from Sigma (St. Louis, MO). Heparinized mouse (non-Swiss albino) plasma was purchased from Lampire Biological Laboratories (Pipersville, PA). Cell culture supplies and media, phosphate-buffered saline (PBS), fetal bovine serum (FBS), sodium pyruvate, non-essential amino acids, and penicillin-streptomycin were obtained from Invitrogen (Carlsbad, CA). Anti-human Cdk2 (M2), Cdk4 (H-22), Cdk6 (C-21), Cyclin D1 (DCS-6), Cyclin E (HE12), Cdc25c (H6), E2F1 (KH95), p21 (C19), and p27 (C19) antibodies were from Santa Cruz Biotechnology, Inc. (Santa Cruz, CA). The anti-human p53 (Ab-6) antibody was from EMD Chemicals, Inc. (Gibbstown, NJ).

### Cell Culture

Human pancreatic cancer cell lines were obtained from the American Type Culture Collection (Rockville, MD). Unless otherwise indicated, all cell culture media contained 10% FBS and 1% penicillin/streptomycin. HPAC cells were grown in a 1∶1 mixture of Dulbecco's modified Eagle's medium and Ham's F12 medium containing 1.2 g/L sodium bicarbonate, 2.5 mM L-glutamine, 15 mM HEPES and 0.5 mM sodium pyruvate supplemented with 2 µg/mL insulin, 5 µg/mL transferrin, 40 ng/mL hydrocortisone, 10 ng/mL epidermal growth factor and 5% fetal bovine serum. Panc-1 cells were cultured with RPMI 1640 containing 1 mM HEPES buffer, 25 µg/mL gentamicin, 1.5 g/L sodium bicarbonate, and 0.25 µg/mL amphotericin B. BxPC3 and Mia Paca-2 cells were grown in RPMI 1640 containing 4.5 g/L glucose and DMEM media, respectively.

### Cell Survival Assay

The effect of the test compound on human pancreatic cancer cell viability, expressed as the percentage of cell survival, was determined using the MTT assay [45]–[47]. Cells were grown in 96-well plates at 4–5×10^3^ cells per well and exposed to different concentrations of the test compound (0, 1, 5, 12.5, 25, 50 or 100 µM). After incubation for different times, 10 µL of the MTT solution (5 mg/mL; Sigma; St. Louis, MO) were added into each well. The plates were incubated for 2–4 hr at 37°C. The supernatant was then removed and the formazan crystals were dissolved with 100 µL of DMSO. The absorbance at 570 nm was recorded using an OPTImax microplate reader (Molecular Devices; Sunnyvale, CA). The cell survival percentages were calculated by dividing the mean optical density (OD) of compound-containing wells by that of DMSO-containing control wells. Three separate experiments were accomplished to determine the IC_50_ values.

### Anchorage-independent Growth Assay

Pancreatic cancer cells were treated with various concentrations of KCN1 in 0.8 ml of 0.35% agar containing 10% FBS. The cultures were maintained in 37°C, 5% CO_2_ incubator for two weeks. Then, cell colonies were observed and scored under a microscope.

### Cell Proliferation Assay

The effect of KCN1 on cell proliferation was determined using the BrdUrd incorporation assay (Oncogene, La Jolla, CA) according to the manufacturer’s instructions [46], [47]. In brief, cells were seeded in 96-well plates (8 × 10^3^ to 1.2 × 10^4^ cells per well) and incubated with various concentrations of KCN1 (0, 5, 12.5 and 50 µM) for 24 h. BrdUrd was added to the medium 10 h before termination of the experiment. The BrdUrd incorporated into cells was determined by anti-BrdUrd antibody, and absorbance was measured at dual wavelengths of 450/540 nm using an OPTImax microplate reader (Molecular Devices; Sunnyvale, CA).

### Detection of Apoptosis

Cells in early and late stages of apoptosis were detected using an Annexin V-FITC apoptosis detection kit from BioVision (Mountain View, CA), according the manufacturer’s protocol [46], [47]. In brief, 2–3×10^5^ cells were exposed to various doses of the test compound KCN1 (0, 5, 12.5, 50 µM) and incubated for 48 h prior to analysis. The samples were analyzed using a Becton Dickinson FACSCalibur instrument (Ex = 488 nm; Em = 530 nm). Cells that were positive for Annexin V-FITC alone (early apoptosis) and Annexin V-FITC and PI (late apoptosis) were counted.

### Cell Cycle Measurements

To determine the effect of KCN1 on the cell cycle, cells (2–3×10^5^) were exposed to the test compounds (0, 5, 12.5 or 50 µM) and incubated for 24 h prior to analysis. Cells were trypsinized, washed with PBS, and fixed in 1.5 mL of 95% ethanol at 4°C overnight, followed by incubation with RNAse and staining with propidium iodide (Sigma). The DNA content was determined by flow cytometry [46], [47].

### Western Blot Analysis

In the *in vitro* studies, cells were exposed to various concentrations of KCN1 for 12 or 24 h. For immunoblotting, cell lysates were fractionated with identical amounts of protein by SDS-PAGE and transferred to Bio-Rad trans-Blot nitrocellulose membranes (Bio-Rad Laboratories, Hercules, CA). The nitrocellulose membrane was then incubated in blocking buffer (Tris-buffered saline containing 0.1% Tween 20 and 5% non-fat milk) for 1 hr at room temperature. Then the membrane was incubated with the appropriate primary antibody overnight at 4°C or 2 h at room temperature with gentle shaking. The membrane was washed three times with rinsing buffer (Tris-buffered saline containing 0.1% Tween 20) for 15 min and then incubated with goat anti-mouse/rabbit IgG-horseradish peroxidase-conjugated antibody (Bio-Rad) for 1 h at room temperature. After repeating the washes in triplicate, the protein of interest was detected by enhanced chemilluminescence reagents from PerkinElmer LAS, Inc (Boston, MA) [46].

### Mouse Xenograft Model of Human Pancreatic Cancer

The animal procedures were performed under approval by the Institutional Animal Use and Care Committee of Texas Tech University Health Sciences Center (IACUC # 10031, PHS Assurance # A 3056-01, USDA Registration # 74-R-0050, REF # 039436). Female athymic pathogen-free nude mice (nu/nu, 4–6 weeks) were purchased from Charles River Laboratories International Inc. (Wilmington, MA). To establish Panc-1 and Mia Paca-2 human pancreatic cancer xenograft tumors, cultured Panc-1 (5×10^6^) and Mia Paca-2 (1×10^7^) cells were harvested from monolayer cultures, washed twice with serum-free medium, re-suspended and injected subcutaneously (total volume 0.2 mL) into the left inguinal area of the mice. All animals were monitored for activity, physical condition, body weight, and tumor growth. Tumor size was determined every other day by caliper measurement of two perpendicular diameters of the implant. Tumor volume (in mm^3^) was calculated by the formula, ½ (*a* × *b* ^2^) where “*a*” is the long diameter and “*b*” is the short diameter [46], [47].

The animals bearing human cancer xenografts were randomly divided into various treatment groups and a control group (7–10 mice/group). The untreated control group received the vehicle only. For the Panc-1 and Mia Paca-2 xenograft models, KCN1 was dissolved in a 1∶1 solution of Cremophor EL (Sigma, St. Louis, MO): ethanol (200 Proof) by heating to 80–90°C in a water bath and vortexing, then diluted with an equal volume of phosphate-buffered saline, and was administered by intraperitoneal injection at doses of 30 and 60 mg/kg/d, 5d/wk for several weeks. Treatment was initiated once tumor volume reached ∼100 mm^3^. The dosage regimen was decided based upon IC_50_ values obtained from *in-vitro* studies and adjustments for body surface and weight of the treated animal.

### Pharmacokinetic Studies in Mice

Female CD-1 and nude mice were administered 35 mg/kg body weight of KCN1 as either a single intravenous injection *via* a tail vein or *via* an intraperitoneal injection. At selected time points after dosing, groups of three mice/time point were subjected to a retro-orbital bleed. The blood was collected into heparinized tubes, and plasma was separated by centrifugation. Tissues (brain, skeletal muscle, kidneys, liver, spleen, and heart) were collected by necropsy. Urine and feces were collected on ice for 24 h from mice housed in metabolic cages (n = 3/study). The collection containers and cages were washed twice with 0.9% saline solution, and each urine collection and each wash was analyzed separately, then values were added together for each mouse to determine the total KCN1 present in urine during the collection period. Pharmacokinetic values were derived using Phoenix WinNonlin Version 6.0 (Mountain View, CA).

### Analytical Methods

An HPLC protocol was developed to detect KCN1 in different matrices (plasma, urine or tissue homogenates) using an Agilent 1120 instrument. KCN1 was separated on a Zorbax SB-C18 (5 µm, 150 × 4.6 mm) analytical column with a Zorbax Reliance Cartridge Guard Column (SB-C18), and the eluate was monitored by UV at 254 nm. The mobile phase was composed of “A” (methanol +0.1% Et_3_N)-“B” (ddH_2_O+0.1% Et_3_N) (80∶20, v/v). Prior to application, the mobile phase was filtered and degassed using a Millipore glass filter system with a nylon membrane (0.2 µm). The peaks for KCN1 were used to establish standard curves and for quantitative analysis of samples. Quantification of the compound was achieved by determination of the peak area at the retention time of approximately 4.4 minutes.

### Treatment of Plasma and Tissue Samples

Plasma (100 µL) was mixed with 200 µL of methanol, vortexed, and centrifuged to remove precipitated proteins. The supernatant was evaporated to dryness under a stream of air. The residue was dissolved in 100 µL of the mobile phase, and 10 µL was injected onto the HPLC system for analysis. Weighed tissues were homogenized in two to five volumes of PBS (based on the weight of the tissue samples), and portions of the homogenates were processed in the same manner as the plasma samples.

### Plasma Stability Determination

The validated HPLC method was used to evaluate the stability of KCN1 in mouse plasma at 37°C, 4°C and −80°C. KCN1 was dissolved in methanol and diluted with plasma to achieve the two concentrations used in the study: 1 and 10 µM. At the designated time points, samples were removed and extracted using the aforementioned procedure. KCN1 concentrations were quantified and, to illustrate the *in vitro* stability of the compound, expressed as percentages of the initial concentration.

### Plasma Protein Binding Assay

The extent to which KCN1 was bound by mouse plasma proteins was assessed by a previously described method involving the use of a micro-ultrafiltration system [48]. Samples of mouse plasma containing KCN1 at concentrations of 1.0 and 10 µM were maintained at 37°C for 1 h. Controls were prepared using methanol in place of plasma. From each of these preparations, a portion was taken and placed in a sample reservoir of an Amicon Centrifree® ultrafiltration system (Millipore Co., Bedford, MA). The filter systems were centrifuged at 2,000 × *g* until the reservoirs were dry. From each sample, triplicate portions were taken for analysis by HPLC. The amounts present in the filtrate were designated as “free drug” (*F*). The concentrations of the unfiltered solutions were also determined by triplicate analyses. This amount represented the “total drug” concentration (*T*). The amount bound to the filter (*X*) was also considered. The percentage of KCN1 bound to plasma proteins was calculated by the following formula: % bound = [(T-F-X)/T]×100.

### Examination of S9 Metabolism

The *in vitro* metabolism of KCN1 was determined using hepatic microsomal S9 fractions from CD2F1 mice (In Vitro Technologies, Boston, MA). The reaction mixture consisted of 20 µL of 1000 µM KCN1 (in DMSO) and 1280 µL of cold 100 mM Tris buffer (pH 7.4). The reactions were initiated by adding 200 µL of the appropriate S9 fraction, and were maintained at 37°C. The negative controls did not contain the hepatic S9 fractions. Metabolic reactions were initiated by adding phase I (NADPH-regenerating systems) or phase II (UDPGA and PAPS) reagents to the reaction mixtures, respectively. At designated time points (0, 15, 30, 45, and 60 min), duplicate 100 µL portions of the incubation mixture were removed and subjected to HPLC analysis by the same procedure described for plasma samples above. The stability of the drug was determined by analysis of intact KCN1, in comparison with negative control (without S9 fractions).

### Data and Statistical Analysis

Experimental data are expressed as means and standard deviations, and the significance of differences was analyzed by ANOVA or Student’s t-test as appropriate.
